# Synthesis and crystal structure of *catena*-poly[[[aqua­(2,3-di­methyl­pyrazine-κ*N*)cadmium(II)]-di-μ-bromido] 2,3-di­methyl­pyrazine monosolvate hemihydrate]

**DOI:** 10.1107/S2056989026005372

**Published:** 2026-05-29

**Authors:** Christian Näther

**Affiliations:** a Institut für Anorganische Chemie, Universität Kiel, Max-Eyth.-Str. 2, 24118 Kiel, Germany; University of Missouri-Columbia, USA

**Keywords:** coordination polymer, synthesis, cadmium bromide, 2,3-di­methyl­pyrazine, crystal structure

## Abstract

Crystals of the title compound, {[CdBr_2_(C_6_H_8_N_2_)(H_2_O)]·C_6_H_8_N_2_·0.5H_2_O}_*n*_ (C_6_H_8_N_2_ = 2,3-di­methyl­pyrazine), were obtained accidentally by the reaction of cadmium bromide with 2,3-di­methyl­pyrazine in water. The asymmetric unit consists of one Cd cation, two bromide anions, one 2,3-di­methyl­pyrazine ligand, one non-coordinating 2,3-di­methyl­pyrazine mol­ecule, and one water mol­ecule in general positions as well as an additional water mol­ecule that is located on a twofold rotation axis. The cadmium cations are octa­hedrally coordinated by four bromide anions, one 2,3-di­methyl­pyrazine ligand and one water mol­ecule, and are linked by pairs of μ-1,1-bridging bromide anions into chains that propagate in the *a*-axis direction. These chains are further connected into layers by inter­molecular hydrogen bonding. The coordinated water ligand is hydrogen bonded to the 2,3-di­methyl­pyrazine, and the water solvate mol­ecule and the 2,3-di­methyl­pyrazine mol­ecules of crystallization are linked by O—H⋯N hydrogen bonding *via* the water solvate mol­ecules into chains along the *c*-axis direction. The water solvate mol­ecule therefore acts as donor for two O—H⋯N and as acceptor for two O—H⋯O hydrogen bonds.

## Abstract 2

In the crystal structure of CdBr_2_(2,3-di­methyl­pyrazine)(H_2_O)-2,3-di­methyl­pyrazine-solvate hemihydrate, the cadmium cations are linked by pairs of μ-1,1 bridging bromide anions into chains that are linked by inter­molecular hydrogen bonding to the water and the 2,3-di­methyl­pyrazine solvate mol­ecules.

## 1. Chemical context

In recent years, numerous transition-metal halide and pseudohalide coordination compounds have been reported. In particular, those with copper(I) show a large structural variability, which can partly be traced back to the fact that these anions can act as bridging anionic ligands, leading to the formation of different metal-halide substructures (Kromp & Sheldrick, 1999 ▸; Peng *et al.*, 2010 ▸; Li *et al.*, 2005 ▸, Näther *et al.*, 2002 ▸). This variability frequently leads to the formation of compounds of different stoichiometry in which the ratio between the metal halide or metal pseudohalide is different. Whether this is the case for a given metal halide or pseudohalide and a given ligand can easily be checked by thermal treatment of coligand-rich compounds because in many cases they lose the neutral ligands in separate steps, leading to the formation of coligand-deficient compounds as inter­mediates (Näther *et al.*, 2001 ▸; Näther & Jess, 2001 ▸).

In the beginning, we focused on compounds based on Cu^I^ but later we also became inter­ested in coordination compounds based on twofold positively charged metal cations such as zinc or cadmium. These compounds are of inter­est, for example, because of their luminescence properties (Mautner *et al.*, 2016 ▸; Jess *et al.*, 2020 ▸). In contrast to copper(I), such compounds do not show a comparable structural variability. However, the structural variability can be enhanced if ligands are used that can act not only as monocoordinating but also as bridging ligands.

In this context, we recently reported on Zn and Cd halide compounds with 2,3-di­methyl­pyrazine as coligand. In contrast to Cd, which frequently exhibits an octa­hedral coordination, Zn cations usually prefer a tetra­hedral coordination, even if compounds with an octa­hedral coordination are known. Compounds of different stoichiometry have been reported with Zn halides and 2,3-di­methyl­pyrazine, including ZnCl_2_(2,3-di­methyl­pyrazine) (Näther & Bhosekar, 2025*a* ▸), ZnBr_2_(2,3-di­methyl­pyrazine) (Näther & Bhosekar, 2025*b* ▸) and ZnI_2_(2,3-di­methyl­pyrazine) (Näther & Bhosekar, 2026 ▸), with a ratio between Zn*X*_2_ and coordinating coligands of 1:1. All of these compounds are isotypic and consist of tetra­hedrally coordinated Zn cations that are linked by the 2,3-di­methyl­pyrazine ligands into chains.

2,3-Di­methyl­pyrazine-rich compounds with a ratio of 1:2 between Zn*X*_2_ and coordinating coligands are also known. These are the isotypic compounds ZnCl_2_(2,3-di­methyl­pyrazine)_2_ (Näther & Bhosekar, 2025*a* ▸) and ZnBr_2_(2,3-di­methyl­pyrazine)_2_ (Yang *et al.*, 2025 ▸), which consist of discrete complexes in which the Zn cations are tetra­hedrally coordinated by two halide anions and two only terminally coordinated 2,3-di­methyl­pyrazine ligands. An additional compound with a ratio of 1:2 is the heteroleptic tetra­hedral discrete complex [ZnI_2_(2,3-di­methyl­pyrazine)(H_2_O)](H_2_O)_0.5_(2,3-di­methyl­pyrazine)_0.5_ that crystallizes with additional water and 2,3-di­methyl­pyrazine as solvate mol­ecules (Näther & Bhosekar, 2026 ▸).

Within this project, we also reported the first compounds with cadmium halides and 2,3-di­methyl­pyrazine, including CdI_2_(2,3-di­methyl­pyrazine)_2_ and CdI_2_(2,3-di­methyl­pyrazine) with a ratio of 1:2 or 1:1 between CdI_2_ and coligands (Näther, 2026 ▸). The former consists of discrete complexes with only terminal coligands, whereas in the latter the 2,3-di­methyl­pyrazine ligands link the Cd cations into chains. These structures are therefore comparable to those with Zn halides and 2,3-di­methyl­pyrazine mentioned above. However, in none of these compounds are condensed metal halide substructures found in which the metal cations are linked by bridging halide anions. Therefore, in the course of our systematic investigations we decided to prepare compounds with cadmium and the remaining halide anions and within these investigations we accidentally obtained crystals from the reaction of CdBr_2_ with 2,3-di­methyl­pyrazine in water that were characterized by single crystal X-ray diffraction.
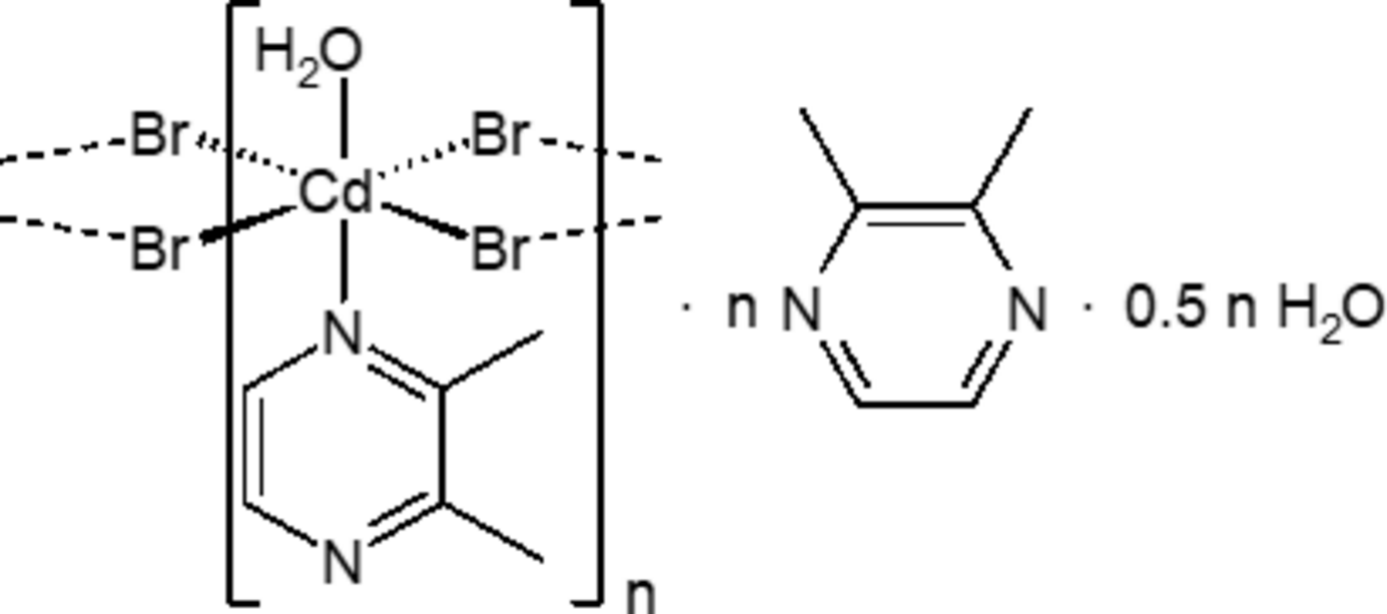


## 2. Structural commentary

**Figure 1:**
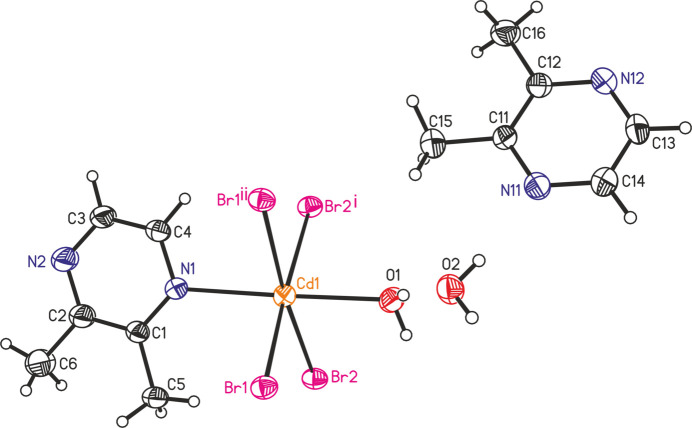
Crystal structure of the title compound with labeling and displacement ellipsoids drawn at the 50% probability level. [Symmetry codes: (i) −*x*, *y*, −*z* + 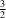; (ii) −*x* + 1, *y*, −*z* + 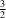.]

The asymmetric unit of the title compound, [CdBr_2_(C_6_H_8_N_2_)(H_2_O)]·C_6_H_8_N_2_·0.5H_2_O (C_6_H_8_N_2_ = 2,3-di­methyl­pyrazine), is built up of a half water mol­ecule that is located on a twofold rotation axis, as well as one cadmium cation, one water mol­ecule, one coordinating and one non-coordinating 2,3-di­methyl­pyrazine mol­ecules that occupy general positions (Fig. 1 ▸). The cadmium cations are sixfold coordinated by four μ-1,1 bridging bromide anions as well as one 2,3-di­methyl­pyrazine ligand and one aqua ligand that occupy the apical positions. The bond lengths deviate only slightly from the ideal values, which shows that the octa­hedra are slightly distorted (Table 1 ▸). Because of steric repulsion, the Br—Cd—Br angles of the bromide atoms in the *cis*-position are larger than 90° (Table 1 ▸).

**Figure 2:**
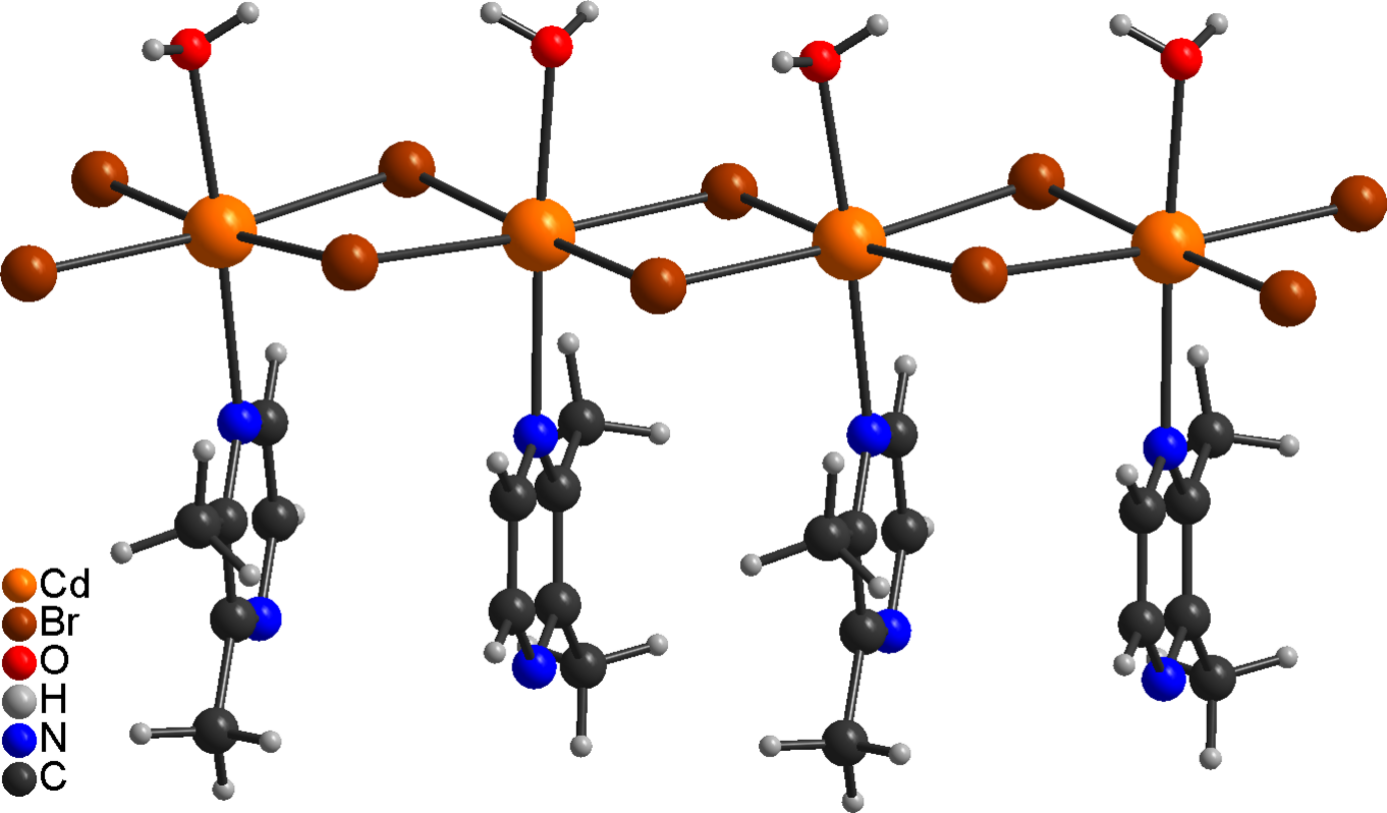
View of part of a chain in the crystal structure of the title compound.

The Cd cations are linked by pairs of μ-1,1-bromide anions into chains along the *a*-axis direction (Fig. 2 ▸). These chains consist of octa­hedra that share common Br edges and the resulting Cd_2_Br_2_ rings are located around a twofold rotation axis. This chain motif is also observed in *catena*-[hexa­kis­(μ_2_-bromo)­diaqua­bis­(2-hy­droxy­ethyl­sulfide-*O*,*S*)tricadmium] (Refcode HAXGUI; Rogers *et al.*, 1993 ▸) and additional examples are given in the *Database survey*.

## 3. Supra­molecular features

**Figure 3:**
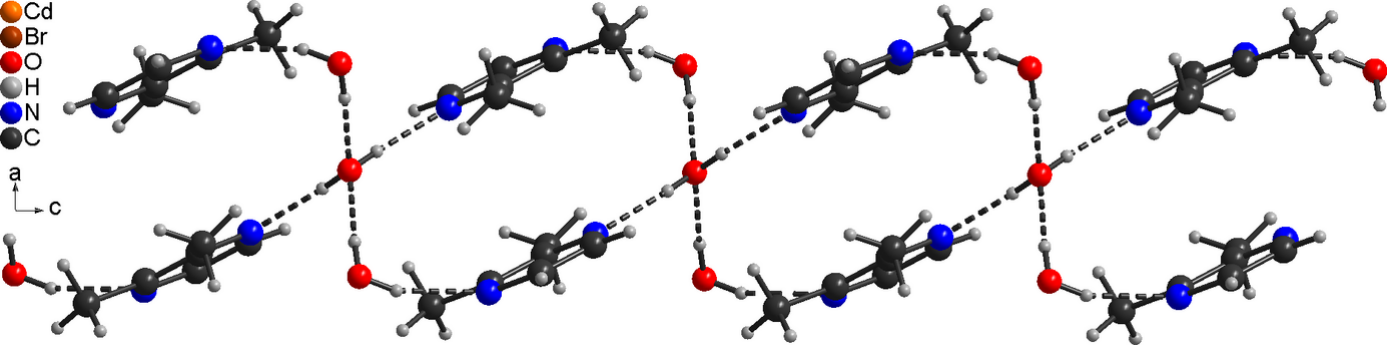
View of the hydrogen-bonded chains in the crystal structure of the title compound with inter­molecular O—H⋯O and O—H⋯N hydrogen bonding shown as dashed lines.

**Figure 4:**
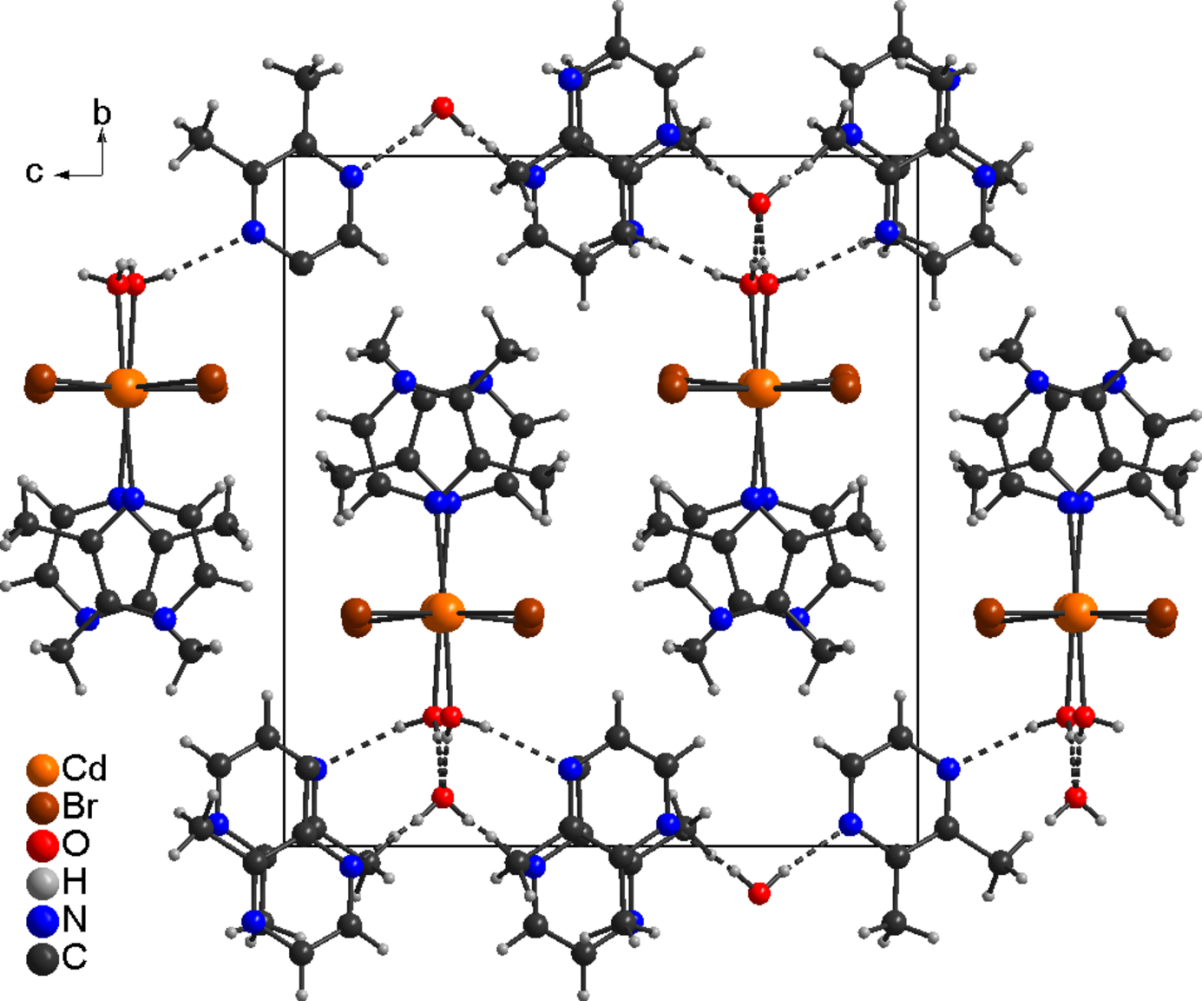
Crystal structure of the title compound in a view along the *a*-axis direction and inter­molecular O—H⋯O and O—H⋯N hydrogen bonding shown as dashed lines.

In the crystal structure of the title compound, the H atoms of the coordinated water mol­ecule are involved in inter­molecular O—H⋯O and O—H⋯N hydrogen bonding to the solvate water mol­ecule and the solvate 2,3-di­methyl­pyrazine mol­ecule (Fig. 3 ▸). The latter is also connected to the solvate water mol­ecule by inter­molecular O—H⋯N hydrogen bonding. The uncoordinated water mol­ecule is involved in four hydrogen bonds. First of all it acts as donor for two O—H⋯N hydrogen bonds to the 2,3-di­methyl­pyrazine mol­ecules (O2—H1*O*2⋯N11) and secondly as acceptor for two O—H⋯O hydrogen bonds to the coordinated water mol­ecules (O1—H1*O*1⋯O2, Fig. 3 ▸). This leads to the formation of hydrogen-bonded chains built up of water and 2,3-di­methyl­pyrazine mol­ecules that propagate in the *c*-axis direction (Fig. 3 ▸). The O—H⋯N and O—H⋯O angles are close to linear, which suggests strong hydrogen-bonding inter­actions (Table 2 ▸). The CdBr_2_ chains and the hydrogen-bonded network condense into layers that are parallel to the *a*/*c*-plane (Fig. 4 ▸). There are additional C—H⋯Br contacts but from the distances and angles they only correspond to weak inter­actions (Table 2 ▸).

## 4. Database survey

As already mentioned in the *Chemical context* section, some compounds with zinc halides and 2,3-di­methyl­pyrazine have already been reported. In contrast, only two cadmium compounds with the composition CdI_2_(2,3-di­methyl­pyrazine)_2_ and CdI_2_(2,3-di­methyl­pyrazine) are known (Näther, 2026 ▸). In both of these compounds the metal cations are in a tetra­hedral coordination and are not connected *via* the halide anions, which, especially for cadmium, is somehow surprising.

However, a search in the CSD (CSD Version 5.43, 2025; Groom *et al.*, 2016 ▸) using CONQUEST (Bruno *et al.*, 2002 ▸) revealed that many compounds with cadmium halides and other pyrazine derivatives as coligands have been reported that also incorporate Cd*X*_2_ chains. These include Cd*X*_2_(pyrazine) [*X* = Cl, Br, I; CSD refcodes TISSUJ (Pickardt & Staub, 1996 ▸), RINSIQ and RINSOW (Bailey & Pennington, 1997 ▸), RINSOW01 and RINSIQ01 (Pickardt & Staub, 1997 ▸)] in which the Cd cations are linked by pairs of bridging halide anions into chains, which are further connected into layers by the pyrazine coligands. Similar Cd*X*_2_ chains are also found in compounds with 2-chloro and 2-methyl­pyrazine (Näther *et al.*, 2017 ▸), including Cd*X*_2_(*L*)_2_ (*X* = Cl, Br, I, *L* = 2-chloro and methyl­pyrazine: QAWHOO, QAWGON, QAWGUT, QAWHAA, QAWHEE and QAWHII). Therefore, in the majority of cases the cadmium cations are linked into chains like those observed in the crystal structure of the title compound.

## 5. Synthesis and crystallization

**General**

Cadmium bromide and 2,3-di­methyl­pyrazine were purchased from Sigma-Aldrich.

**Synthesis of the title compound**

1 mmol (272.2 mg) of CdBr_2_ and 2.0 mmol (216.3 mg) of 2,3-di­methyl­pyrazine were reacted in 3 mL of water for 3 d at room temperature, which led to the formation of crystals suitable for single-crystal X-ray diffraction analysis.

## 6. Refinement

Crystal data, data collection and structure refinement details are summarized in Table 3 ▸. The C—H hydrogen atoms were positioned with idealized geometry (methyl H atoms allowed to rotate but not to tip) and were refined isotropically with *U*_iso_(H) = 1.2 *U*_eq_(C) (1.5 for methyl H atoms). The O—H hydrogen atoms were located in difference maps, their bond lengths were set to ideal values and finally they were refined isotropically with *U*_iso_(H) = 1.5 *U*_eq_(O) using a riding model.

## Supplementary Material

Crystal structure: contains datablock(s) I. DOI: 10.1107/S2056989026005372/ev2028sup1.cif

Structure factors: contains datablock(s) I. DOI: 10.1107/S2056989026005372/ev2028Isup2.hkl

CCDC reference: 2555772

Additional supporting information:  crystallographic information; 3D view; checkCIF report

## Figures and Tables

**Table 1:** Selected geometric parameters (Å, °)

Cd1—O1	2.346 (2)	Cd1—Br2	2.7255 (4)
Cd1—N1	2.484 (3)	Cd1—Br2^i^	2.7450 (4)
Cd1—Br1	2.7245 (4)	Cd1—Br1^ii^	2.7497 (4)
			
O1—Cd1—N1	176.18 (8)	Br2—Cd1—Br2^i^	90.562 (11)
O1—Cd1—Br1	87.46 (6)	O1—Cd1—Br1^ii^	91.94 (6)
N1—Cd1—Br1	96.11 (6)	N1—Cd1—Br1^ii^	89.49 (6)
O1—Cd1—Br2	83.45 (6)	Br1—Cd1—Br1^ii^	88.437 (11)
N1—Cd1—Br2	95.00 (6)	Br2—Cd1—Br1^ii^	175.052 (12)
Br1—Cd1—Br2	93.156 (11)	Br2^i^—Cd1—Br1^ii^	87.487 (11)
O1—Cd1—Br2^i^	88.39 (6)	Cd1—Br1—Cd1^ii^	90.863 (11)
N1—Cd1—Br2^i^	88.14 (6)	Cd1—Br2—Cd1^i^	89.438 (11)
Br1—Cd1—Br2^i^	174.086 (12)		

Symmetry codes: (i) 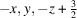; (ii) 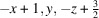.

**Table 2:** Hydrogen-bond geometry (Å, °)

*D*—H⋯*A*	*D*—H	H⋯*A*	*D*⋯*A*	*D*—H⋯*A*
O1—H1*O*1⋯O2	0.84	2.01	2.839 (3)	169
O1—H2*O*1⋯N12^iii^	0.84	2.13	2.926 (4)	158
C4—H4⋯Br1^ii^	0.95	3.00	3.592 (3)	122
C4—H4⋯Br2^i^	0.95	3.01	3.545 (3)	117
C5—H5*A*⋯Br1	0.98	2.94	3.804 (3)	147
C5—H5*A*⋯Br2	0.98	3.01	3.657 (4)	125
C15—H15*C*⋯O2	0.98	2.65	3.449 (4)	139
O2—H1*O*2⋯N11	0.84	2.02	2.861 (3)	174

Symmetry codes: (i) 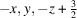; (ii) 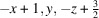; (iii) 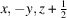.

**Table 3:** Experimental details

Crystal data	
Chemical formula	[CdBr_2_(C_6_H_8_N_2_)(H_2_O)]·C_6_H_8_N_2_·0.5H_2_O
*M*_r_	515.53
Crystal system, space group	Monoclinic, *P*2/*c*
Temperature (K)	170
*a*, *b*, *c* (Å)	7.7459 (4), 15.4368 (5), 14.1867 (7)
β (°)	90.621 (4)
*V* (Å^3^)	1696.23 (13)
*Z*	4
Radiation type	Mo *K*α
μ (mm^−1^)	6.00
Crystal size (mm)	0.13 × 0.11 × 0.09

Data collection	
Diffractometer	Stoe IPDS2
Absorption correction	Numerical (*X-RED* and *X-SHAPE*; Stoe, 2002 ▸)
*T*_min_, *T*_max_	0.338, 0.482
No. of measured, independent and observed [*I* > 2σ(*I*)] reflections	13656, 4097, 3340
*R*_int_	0.031
(sin θ/λ)_max_ (Å^−1^)	0.661

Refinement	
*R*[*F*^2^ > 2σ(*F*^2^)], *wR*(*F*^2^), *S*	0.033, 0.089, 1.04
No. of reflections	4097
No. of parameters	191
H-atom treatment	H-atom parameters constrained
Δρ_max_, Δρ_min_ (e Å^−3^)	0.74, −0.63

Computer programs: *X-AREA* (Stoe, 2002 ▸), *SHELXT2014/4* (Sheldrick, 2015*b* ▸), *SHELXL2016/6* (Sheldrick, 2015*a* ▸), *DIAMOND* (Brandenburg, 1999 ▸), *XP* in *SHELXTL-PC* (Sheldrick, 2008 ▸) and *publCIF* (Westrip, 2010 ▸).

## supplementary crystallographic information

### catena-Poly[[[aqua(2,3-dimethylpyrazine-κN)cadmium(II)]-di-µ-bromido] 2,3-dimethylpyrazine monosolvate hemihydrate] Crystal data

**Table:**

[CdBr_2_(C_6_H_8_N_2_)(H_2_O)]·C_6_H_8_N_2_·0.5H_2_O	*F*(000) = 996
*M**_r_* = 515.53	*D*_x_ = 2.019 Mg m^−^^3^
Monoclinic, *P*2/*c*	Mo *K*α radiation, λ = 0.71073 Å
*a* = 7.7459 (4) Å	Cell parameters from 12616 reflections
*b* = 15.4368 (5) Å	θ = 2.5–27.3°
*c* = 14.1867 (7) Å	µ = 6.00 mm^−^^1^
β = 90.621 (4)°	*T* = 170 K
*V* = 1696.23 (13) Å^3^	Block, colorless
*Z* = 4	0.13 × 0.11 × 0.09 mm

### catena-Poly[[[aqua(2,3-dimethylpyrazine-κN)cadmium(II)]-di-µ-bromido] 2,3-dimethylpyrazine monosolvate hemihydrate] Data collection

**Table:**

Stoe IPDS-2 diffractometer	3340 reflections with *I* > 2σ(*I*)
ω scans	*R*_int_ = 0.031
Absorption correction: numerical (X-Red and X-Shape; Stoe, 2002)	θ_max_ = 28.0°, θ_min_ = 2.0°
*T*_min_ = 0.338, *T*_max_ = 0.482	*h* = −10→9
13656 measured reflections	*k* = −20→20
4097 independent reflections	*l* = −18→18

### catena-Poly[[[aqua(2,3-dimethylpyrazine-κN)cadmium(II)]-di-µ-bromido] 2,3-dimethylpyrazine monosolvate hemihydrate] Refinement

**Table:**

Refinement on *F*^2^	Hydrogen site location: mixed
Least-squares matrix: full	H-atom parameters constrained
*R*[*F*^2^ > 2σ(*F*^2^)] = 0.033	*w* = 1/[σ^2^(*F*_o_^2^) + (0.0537*P*)^2^] where *P* = (*F*_o_^2^ + 2*F*_c_^2^)/3
*wR*(*F*^2^) = 0.089	(Δ/σ)_max_ = 0.001
*S* = 1.04	Δρ_max_ = 0.74 e Å^−^^3^
4097 reflections	Δρ_min_ = −0.63 e Å^−^^3^
191 parameters	Extinction correction: SHELXL-2016/6 (Sheldrick 2016), Fc^*^=kFc[1+0.001xFc^2^λ^3^/sin(2θ)]^-1/4^
0 restraints	Extinction coefficient: 0.0028 (3)

### catena-Poly[[[aqua(2,3-dimethylpyrazine-κN)cadmium(II)]-di-µ-bromido] 2,3-dimethylpyrazine monosolvate hemihydrate] Special details

**Table:**

Geometry. All esds (except the esd in the dihedral angle between two l.s. planes) are estimated using the full covariance matrix. The cell esds are taken into account individually in the estimation of esds in distances, angles and torsion angles; correlations between esds in cell parameters are only used when they are defined by crystal symmetry. An approximate (isotropic) treatment of cell esds is used for estimating esds involving l.s. planes.

### catena-Poly[[[aqua(2,3-dimethylpyrazine-κN)cadmium(II)]-di-µ-bromido] 2,3-dimethylpyrazine monosolvate hemihydrate] Fractional atomic coordinates and isotropic or equivalent isotropic displacement parameters (Å^2^)

**Table:**

	*x*	*y*	*z*	*U*_iso_*/*U*_eq_	
Cd1	0.24844 (3)	0.33730 (2)	0.75394 (2)	0.02363 (9)	
Br1	0.50745 (4)	0.32344 (2)	0.88453 (2)	0.02634 (10)	
Br2	−0.00279 (4)	0.33703 (2)	0.88698 (2)	0.02708 (10)	
O1	0.2182 (3)	0.18633 (15)	0.76353 (15)	0.0309 (5)	
H1O1	0.309445	0.157649	0.756830	0.046*	
H2O1	0.181245	0.172289	0.816830	0.046*	
N1	0.2606 (3)	0.49752 (17)	0.73937 (17)	0.0261 (5)	
C1	0.2571 (4)	0.5595 (2)	0.8054 (2)	0.0275 (6)	
C2	0.2539 (5)	0.6471 (2)	0.7793 (3)	0.0388 (8)	
N2	0.2579 (6)	0.67206 (19)	0.6891 (2)	0.0483 (9)	
C3	0.2627 (5)	0.6087 (2)	0.6244 (2)	0.0389 (8)	
H3	0.265776	0.623769	0.559538	0.047*	
C4	0.2633 (4)	0.5231 (2)	0.6493 (2)	0.0309 (7)	
H4	0.265691	0.480448	0.601080	0.037*	
C5	0.2568 (5)	0.5342 (2)	0.9072 (2)	0.0331 (7)	
H5A	0.274417	0.471500	0.912769	0.050*	
H5B	0.145650	0.549789	0.934936	0.050*	
H5C	0.349968	0.564623	0.940715	0.050*	
C6	0.2480 (8)	0.7174 (3)	0.8526 (3)	0.0617 (13)	
H6A	0.243977	0.774031	0.821427	0.093*	
H6B	0.351389	0.713953	0.892862	0.093*	
H6C	0.144923	0.710028	0.891199	0.093*	
N11	0.3329 (4)	−0.03052 (18)	0.60583 (19)	0.0325 (6)	
C11	0.2779 (4)	0.0162 (2)	0.5316 (2)	0.0273 (6)	
C12	0.2007 (4)	−0.0250 (2)	0.4535 (2)	0.0288 (6)	
N12	0.1791 (4)	−0.11062 (19)	0.4512 (2)	0.0331 (6)	
C13	0.2314 (5)	−0.1561 (2)	0.5267 (2)	0.0345 (7)	
H13	0.215947	−0.217148	0.527435	0.041*	
C14	0.3067 (5)	−0.1161 (2)	0.6029 (2)	0.0357 (7)	
H14	0.341562	−0.150324	0.655451	0.043*	
C15	0.3008 (5)	0.1122 (2)	0.5364 (3)	0.0384 (8)	
H15A	0.191047	0.139386	0.553252	0.058*	
H15B	0.337665	0.133830	0.474869	0.058*	
H15C	0.388631	0.126313	0.584184	0.058*	
C16	0.1419 (5)	0.0250 (2)	0.3681 (2)	0.0393 (8)	
H16A	0.242693	0.047585	0.334999	0.059*	
H16B	0.068520	0.073317	0.387805	0.059*	
H16C	0.076088	−0.013282	0.325975	0.059*	
O2	0.500000	0.0696 (2)	0.750000	0.0333 (7)	
H1O2	0.450360	0.037064	0.710700	0.050*	

### catena-Poly[[[aqua(2,3-dimethylpyrazine-κN)cadmium(II)]-di-µ-bromido] 2,3-dimethylpyrazine monosolvate hemihydrate] Atomic displacement parameters (Å^2^)

**Table:**

	*U*^11^	*U*^22^	*U*^33^	*U*^12^	*U*^13^	*U*^23^
Cd1	0.02233 (14)	0.02422 (14)	0.02434 (14)	0.00021 (7)	−0.00011 (9)	0.00123 (7)
Br1	0.02315 (16)	0.03242 (17)	0.02344 (16)	−0.00015 (12)	0.00002 (11)	0.00176 (11)
Br2	0.02306 (17)	0.03493 (19)	0.02322 (16)	0.00152 (11)	−0.00020 (11)	−0.00067 (11)
O1	0.0348 (11)	0.0290 (11)	0.0290 (10)	0.0023 (10)	0.0047 (9)	0.0027 (9)
N1	0.0307 (13)	0.0250 (13)	0.0228 (12)	−0.0010 (10)	−0.0005 (10)	0.0017 (9)
C1	0.0331 (16)	0.0260 (15)	0.0234 (14)	0.0010 (12)	0.0013 (12)	−0.0024 (11)
C2	0.060 (2)	0.0293 (17)	0.0274 (15)	0.0017 (17)	0.0016 (14)	−0.0009 (13)
N2	0.088 (3)	0.0268 (16)	0.0302 (15)	0.0007 (16)	0.0021 (16)	0.0029 (11)
C3	0.064 (2)	0.0276 (17)	0.0247 (16)	0.0019 (16)	0.0002 (15)	0.0026 (12)
C4	0.0394 (17)	0.0300 (16)	0.0231 (14)	0.0001 (13)	−0.0009 (12)	−0.0023 (12)
C5	0.0446 (19)	0.0325 (17)	0.0221 (14)	0.0006 (15)	0.0002 (13)	−0.0016 (12)
C6	0.120 (4)	0.0265 (18)	0.038 (2)	0.004 (2)	0.006 (2)	−0.0026 (15)
N11	0.0389 (15)	0.0280 (14)	0.0306 (13)	0.0007 (12)	−0.0047 (11)	−0.0005 (10)
C11	0.0308 (16)	0.0254 (14)	0.0257 (14)	−0.0002 (12)	0.0034 (12)	0.0008 (11)
C12	0.0298 (15)	0.0297 (16)	0.0267 (14)	−0.0004 (12)	0.0007 (12)	−0.0003 (12)
N12	0.0388 (15)	0.0295 (14)	0.0311 (14)	−0.0016 (12)	0.0014 (12)	−0.0039 (11)
C13	0.0411 (19)	0.0232 (15)	0.0393 (17)	0.0004 (13)	0.0001 (14)	−0.0024 (13)
C14	0.0426 (19)	0.0299 (17)	0.0345 (17)	0.0011 (15)	−0.0012 (14)	0.0030 (13)
C15	0.048 (2)	0.0286 (17)	0.0382 (18)	−0.0039 (15)	−0.0019 (16)	−0.0008 (13)
C16	0.049 (2)	0.0388 (19)	0.0304 (16)	0.0063 (16)	−0.0018 (15)	0.0012 (14)
O2	0.0354 (17)	0.0290 (17)	0.0353 (17)	0.000	−0.0078 (14)	0.000

### catena-Poly[[[aqua(2,3-dimethylpyrazine-κN)cadmium(II)]-di-µ-bromido] 2,3-dimethylpyrazine monosolvate hemihydrate] Geometric parameters (Å, º)

**Table:**

Cd1—O1	2.346 (2)	C6—H6A	0.9800
Cd1—N1	2.484 (3)	C6—H6B	0.9800
Cd1—Br1	2.7245 (4)	C6—H6C	0.9800
Cd1—Br2	2.7255 (4)	N11—C14	1.337 (4)
Cd1—Br2^i^	2.7450 (4)	N11—C11	1.342 (4)
Cd1—Br1^ii^	2.7497 (4)	C11—C12	1.406 (5)
O1—H1O1	0.8400	C11—C15	1.493 (5)
O1—H2O1	0.8400	C12—N12	1.332 (4)
N1—C4	1.338 (4)	C12—C16	1.503 (5)
N1—C1	1.339 (4)	N12—C13	1.340 (5)
C1—C2	1.402 (5)	C13—C14	1.370 (5)
C1—C5	1.497 (4)	C13—H13	0.9500
C2—N2	1.337 (4)	C14—H14	0.9500
C2—C6	1.504 (5)	C15—H15A	0.9800
N2—C3	1.342 (4)	C15—H15B	0.9800
C3—C4	1.368 (5)	C15—H15C	0.9800
C3—H3	0.9500	C16—H16A	0.9800
C4—H4	0.9500	C16—H16B	0.9800
C5—H5A	0.9800	C16—H16C	0.9800
C5—H5B	0.9800	O2—H1O2	0.8400
C5—H5C	0.9800	O2—H1O2^ii^	0.8400
			
O1—Cd1—N1	176.18 (8)	H5A—C5—H5B	109.5
O1—Cd1—Br1	87.46 (6)	C1—C5—H5C	109.5
N1—Cd1—Br1	96.11 (6)	H5A—C5—H5C	109.5
O1—Cd1—Br2	83.45 (6)	H5B—C5—H5C	109.5
N1—Cd1—Br2	95.00 (6)	C2—C6—H6A	109.5
Br1—Cd1—Br2	93.156 (11)	C2—C6—H6B	109.5
O1—Cd1—Br2^i^	88.39 (6)	H6A—C6—H6B	109.5
N1—Cd1—Br2^i^	88.14 (6)	C2—C6—H6C	109.5
Br1—Cd1—Br2^i^	174.086 (12)	H6A—C6—H6C	109.5
Br2—Cd1—Br2^i^	90.562 (11)	H6B—C6—H6C	109.5
O1—Cd1—Br1^ii^	91.94 (6)	C14—N11—C11	117.4 (3)
N1—Cd1—Br1^ii^	89.49 (6)	N11—C11—C12	120.2 (3)
Br1—Cd1—Br1^ii^	88.437 (11)	N11—C11—C15	117.5 (3)
Br2—Cd1—Br1^ii^	175.052 (12)	C12—C11—C15	122.3 (3)
Br2^i^—Cd1—Br1^ii^	87.487 (11)	N12—C12—C11	121.4 (3)
Cd1—Br1—Cd1^ii^	90.863 (11)	N12—C12—C16	116.9 (3)
Cd1—Br2—Cd1^i^	89.438 (11)	C11—C12—C16	121.7 (3)
Cd1—O1—H1O1	115.6	C12—N12—C13	117.6 (3)
Cd1—O1—H2O1	110.1	N12—C13—C14	121.2 (3)
H1O1—O1—H2O1	105.2	N12—C13—H13	119.4
C4—N1—C1	117.2 (3)	C14—C13—H13	119.4
C4—N1—Cd1	112.0 (2)	N11—C14—C13	122.2 (3)
C1—N1—Cd1	130.7 (2)	N11—C14—H14	118.9
N1—C1—C2	120.3 (3)	C13—C14—H14	118.9
N1—C1—C5	119.3 (3)	C11—C15—H15A	109.5
C2—C1—C5	120.4 (3)	C11—C15—H15B	109.5
N2—C2—C1	122.0 (3)	H15A—C15—H15B	109.5
N2—C2—C6	117.0 (3)	C11—C15—H15C	109.5
C1—C2—C6	120.9 (3)	H15A—C15—H15C	109.5
C2—N2—C3	116.5 (3)	H15B—C15—H15C	109.5
N2—C3—C4	121.8 (3)	C12—C16—H16A	109.5
N2—C3—H3	119.1	C12—C16—H16B	109.5
C4—C3—H3	119.1	H16A—C16—H16B	109.5
N1—C4—C3	122.1 (3)	C12—C16—H16C	109.5
N1—C4—H4	118.9	H16A—C16—H16C	109.5
C3—C4—H4	118.9	H16B—C16—H16C	109.5
C1—C5—H5A	109.5	H1O2—O2—H1O2^ii^	106.7
C1—C5—H5B	109.5		

Symmetry codes: (i) −*x*, *y*, −*z*+3/2; (ii) −*x*+1, *y*, −*z*+3/2.

### catena-Poly[[[aqua(2,3-dimethylpyrazine-κN)cadmium(II)]-di-µ-bromido] 2,3-dimethylpyrazine monosolvate hemihydrate] Hydrogen-bond geometry (Å, º)

**Table:**

*D*—H···*A*	*D*—H	H···*A*	*D*···*A*	*D*—H···*A*
O1—H1*O*1···O2	0.84	2.01	2.839 (3)	169
O1—H2*O*1···N12^iii^	0.84	2.13	2.926 (4)	158
C4—H4···Br1^ii^	0.95	3.00	3.592 (3)	122
C4—H4···Br2^i^	0.95	3.01	3.545 (3)	117
C5—H5*A*···Br1	0.98	2.94	3.804 (3)	147
C5—H5*A*···Br2	0.98	3.01	3.657 (4)	125
C15—H15*C*···O2	0.98	2.65	3.449 (4)	139
O2—H1*O*2···N11	0.84	2.02	2.861 (3)	174

Symmetry codes: (i) −*x*, *y*, −*z*+3/2; (ii) −*x*+1, *y*, −*z*+3/2; (iii) *x*, −*y*, *z*+1/2.

## References

[bb24] Bailey, R. D. & Pennington, W. T. (1997). *Polyhedron*, **16**, 417–422.

[bb1] Brandenburg, K. (1999). *DIAMOND*. Crystal Impact GbR, Bonn, Germany.

[bb2] Bruno, I. J., Cole, J. C., Edgington, P. R., Kessler, M., Macrae, C. F., McCabe, P., Pearson, J. & Taylor, R. (2002). *Acta Cryst.* B**58**, 389–397. 10.1107/s0108768102003324 12037360

[bb3] Groom, C. R., Bruno, I. J., Lightfoot, M. P. & Ward, S. C. (2016). *Acta Cryst*. B**72**, 171–179. 10.1107/S2052520616003954 PMC4822653 27048719

[bb4] Jess, I., Neumann, T., Terraschke, H., Gallo, G., Dinnebier, R. & Näther, C. (2020). *Z. Anorg. Allg. Chem.* **646**, 1046–1054.

[bb5] Kromp, T. & Sheldrick, W. S. (1999). *Z. Naturforsch. B* **54**, 1175–1180.

[bb6] Li, D., Shi, W. J. & Hou, L. (2005). *Inorg. Chem.* **44**, 3907–3913. 10.1021/ic050209j 15907117

[bb7] Mautner, F. A., Berger, C., Fischer, R. C. & Massoud, S. S. (2016). *Inorg. Chim. Acta* **439**, 69–76.

[bb8] Näther, C. (2026). *Acta Cryst.* E**82**, 394–399. 10.1107/S2056989026002896 PMC13055956 41953346

[bb9] Näther, C. & Bhosekar, G. (2025*a*). *Acta Cryst.* E**81**, 694–698. 10.1107/S205698902500619X PMC12326493 40777540

[bb10] Näther, C. & Bhosekar, G. (2025*b*). *Acta Cryst.* E**81**, 928–931. 10.1107/S2056989025007613 PMC12498051 41059312

[bb11] Näther, C. & Bhosekar, G. (2026). *Acta Cryst.* E**82**, 244–248. 10.1107/S2056989026001088 PMC12961651 41799052

[bb12] Näther, C., Greve, J. & Jess, I. (2002). *Solid State Sci.* **4**, 813–820.

[bb13] Näther, C., Jess, I. & Greve, J. (2001). *Polyhedron* **20**, 1017–1022.

[bb14] Näther, C. & Jess, I. (2001). *Monatsh. Chem.* **132**, 897–910.

[bb35] Näther, C., Jess, I., Germann, L. S., Dinnebier, R. E., Braun, M. & Terraschke, H. (2017). *Eur. J. Inorg. Chem.* pp. 1245–1255.

[bb15] Peng, R., Li, M. & Li, D. (2010). *Coord. Chem. Rev.* **254**, 1–18.

[bb36] Pickardt, J. & Staub, B. (1996). *Z. Naturforsch* B**51**, 947–951.

[bb16] Pickardt, J. & Staub, B. (1997). *Z. Naturforsch* B**52**, 1456–1460.

[bb17] Rogers, R. D., Bond, A. H. & Aguinaga, S. (1993). *J. Crystallogr. Spectrosc. Res.* **23**, 657–661.

[bb18] Sheldrick, G. M. (2008). *Acta Cryst.* A**64**, 112–122. 10.1107/S0108767307043930 18156677

[bb19] Sheldrick, G. M. (2015*a*). *Acta Cryst.* A**71**, 3–8.

[bb20] Sheldrick, G. M. (2015*b*). *Acta Cryst.* C**71**, 3–8.

[bb21] Stoe (2002). *X-AREA*, *X-RED* and *X-SHAPE*. Stoe & Cie, Darmstadt, Germany.

[bb22] Westrip, S. P. (2010). *J. Appl. Cryst.* **43**, 920–925.

[bb23] Yang, C., Zheng, J., Xu, C., Xiao, C., Chang, Y., Zhou, L. & Gong, X. (2025). *Chem. Commun.* **61**, 4379–4382. 10.1039/d4cc06675h 39989335

